# Evaluation of Awareness, Use, and Perceptions of Injury Prevention Programs Among Youth Sport Coaches in Poland

**DOI:** 10.3390/jcm14144951

**Published:** 2025-07-12

**Authors:** Bartosz Wilczyński, Patryk Szczurowski, Jakub Hinca, Łukasz Radzimiński, Katarzyna Zorena

**Affiliations:** 1 Department of Immunobiology and Environmental Microbiology, Medical University of Gdansk, 80211 Gdansk, Poland; katarzyna.zorena@gumed.edu.pl; 2 Gdansk College of Health, 80335 Gdansk, Poland; patryk.szczurowski4@wp.pl; 3 Department of Physical Culture, Gdansk University of Physical Education and Sport, 80336 Gdansk, Poland; jakub.hinca@awf.gda.pl; 4 Department of Physiology and Biochemistry, Gdansk University of Physical Education and Sport, 80336 Gdansk, Poland; lukasz.radziminski@awf.gda.pl

**Keywords:** injury prevention, youth sport, sports injuries, coaching, health promotion, curriculum, coach education

## Abstract

**Background/Objectives**: Injury prevention programs (IPPs) are evidence-based interventions that reduce musculoskeletal injuries in youth sports. Despite their proven benefits, the adoption of IPPs by coaches remains limited. This study aimed to evaluate the awareness, usage, and perceptions of IPPs among youth sports coaches in Poland and to identify factors associated with their implementation. **Methods**: A cross-sectional study was conducted using a web-based survey tailored to youth sports coaches in Poland. Coaches of athletes aged 9–17 were recruited through targeted outreach to clubs and professional networks. The survey assessed IPP awareness, implementation, perceptions, and sources of information. Statistical analyses included chi-square tests, non-parametric comparisons, Firth’s logistic regression, and cluster profiling. **Results**: Only 54.6% of coaches (59 out of 108) were aware of IPPs, and among them, just 47.5% reported using them. No significant associations were found between IPP use and demographic variables such as gender, sport, or place of residence. Coaches who were aware of IPPs were significantly younger than those who were unaware (*p* = 0.029). The information source was the strongest predictor of IPP implementation: coaches trained via formal courses were over 20 times more likely to use IPPs compared to those learning from peers (OR = 20.4, *p* < 0.001). While coaches generally perceived IPPs as beneficial for fitness and recovery, 28.6% expressed doubts about their effectiveness in reducing injury risk. **Conclusions**: Despite broadly positive beliefs, only 47.5% of coaches who were aware of IPPs reported using them. Formal training significantly enhances the likelihood of adoption. These findings emphasize the need for structured educational efforts and improved dissemination strategies to promote evidence-based injury prevention in youth sports settings.

## 1. Introduction

Injury prevention programs (IPPs) play a crucial component in reducing the risk of musculoskeletal injuries, which are a major concern in youth sports [1]. Several evidence-based programs such as FIFA 11+ (and 11+ Kids) [2], OSTRC [3], VolleyVeilig [4], Knee Control+ [5], and Activate [6] have been developed and validated across various team sports. These programs have demonstrated efficacy in reducing lower limb injury rates when consistently applied. As emphasized in recent studies, effective IPPs should be evidence-based, widely disseminated, routinely implemented, and continuously evaluated for long-term sustainability [7,8,9].

Despite strong scientific support, the real-world implementation of IPPs remains limited in youth sports settings [10,11,12]. Coaches have a central role in the implementation of these programs through their authority, education, management, and role in conducting daily training. However, research has shown that awareness of IPPs was inconsistent [12] or low in specific IPPs such as FIFA 11+ [7,13]. The percentage of youth football coaches who use some form of IPP ranges from 22% to 31% [10,13,14,15]. A study conducted among grassroots soccer coaches in Europe revealed that only 16% have knowledge about IPPs; however, 100% of those believe that IPPs are important for players [14]. A cross-sectional study by Donaldson et al., 2018, revealed that almost half (42%) of the coaches were unaware of the FIFA 11+ program, and only one-third (31%) reported using it [7].

Although the injury-reducing and motor skills benefits of IPPs are well-documented [12,16], their successful implementation by coaches is hindered by a variety of factors. The lack of proper training for coaches, time, and resources further hinders the consistent application of these programs in real-world settings [12]. A study on European coaches highlighted a need for better educational resources and training frameworks [14]. Similarly, youth soccer coaches in the United States expressed interest in using IPPs but cited a lack of time and access to proper demonstrations as barriers and were more likely to use a IPP if they had completed one or more educational courses [13].

In addition to knowledge and training, coaches’ perceptions and beliefs play a critical role in IPP adoption [12]. A recent systematic review found that while most coaches had positive attitudes regarding IPPs and believed that they would reduce the risk of injury, others wanted assurance that the programs would improve performance [12]. Beliefs, knowledge, and sources of information may therefore shape whether and how coaches use these programs.

Given potential cultural and educational differences in Poland—including language barriers and limited IPP access—the knowledge and implementation patterns observed elsewhere may not fully apply. Therefore, the purpose of this study was to examine the awareness, use, and perceptions of IPPs among Polish sports coaches working with youth athletes. A secondary objective was to assess the associations between demographic characteristics and sources of information related to IPPs. We hypothesized that (1) awareness and implementation would be moderate to low; (2) formal education or structured training would be positively associated with IPP usage; and (3) coaches’ beliefs about program benefits would vary depending on their information sources. Understanding coaches’ awareness, implementation practices, and beliefs is critical for improving the dissemination of IPPs and enhancing the safety of young athletes.

## 2. Materials and Methods

### 2.1. Participants

The authors’ survey questionnaire aimed to recruit coaches of youth sports groups comprising participants ranging from 9 to 17 years of age. The recruitment process involved emailing a link to the survey directly to coaches and sports clubs, as well as sharing the link on social media platforms in forums and groups specifically intended for sports coaches. This approach constituted a non-probability, convenience sampling strategy based on voluntary participation. Participants included in the study were required to meet the following inclusion criteria: (i) being aged 18 years or above (ii) currently coaching youth athletes aged 9–17 years; and (iii) being actively involved in sports training during the period of the study. A total of 108 respondents participated in this study. The sample included 76 male (70.4%) and 32 female (29.6%) coaches, with a mean age of 33.1 years (SD = 8.6). Most participants resided in cities (88.9%), and the most frequently represented sports were football (41.7%), volleyball (14.8%), handball (12.0%), and basketball (12.0%).

This quantitative study utilized a web-based survey to explore practitioners’ current knowledge, use, and perceptions regarding IPPs within their respective sports. Data collection occurred online over a five-month period, from February to June 2024. The respondents represented various sports disciplines, i.e., football, handball, volleyball, and basketball. The study was part of a research project and w\as approved in advance by the Independent Bioethics Committee (application number: NKBBN/241/2023). All data were gathered through an online survey that included a mix of multiple-choice and open-ended questions. During the preparation of this manuscript, the authors used ChatGPT-4o (OpenAI, San Francisco, CA, USA) and Claude Sonnet 4 (Anthropic, San Francisco, CA, USA) for the editing of English grammar. These tools were not used to generate scientific content, analyze data, or interpret findings. The authors have reviewed and edited the output and take full responsibility for the content of this publication.

### 2.2. Survey Instrument

The questionnaire consisted of 13 items and is provided in full in Table A2. Face and content validity were established through expert consultation and pilot testing with two sports coaches and two sports scientists. Refinements were made to ensure clarity, relevance, and alignment with the current injury prevention literature. The final version was reviewed by the research team, which included experts in team sports, coaching education, and injury prevention research. The questionnaire included a combination of single-choice, multiple-choice, Likert-scale, and open-ended items to ensure comprehensive data capture on both factual practices and subjective perceptions. The questionnaire was organized into the following sections:

(i) Coaching profile—Participants identified the primary sport they coached (e.g., football, volleyball, basketball, etc.) using a one-choice item with an open “Other” field (Q6).

(ii) Awareness (Knowledge) of IPPs—Coaches were asked whether they were familiar with IPPs for the musculoskeletal system (Q7) and how they learned about them. Response options included training/workshops, social media, scientific articles, magazines, and coaching peers, with an open-ended “Other” option (Q8). Respondents could select multiple options but were asked to indicate one primary source.

(iii) Implementation practices—Respondents were asked whether they use IPPs in their teams (Q9), and if so, which specific programs (e.g., FIFA 11+, OSTRC, PEP, Touchfit360, etc.) they use (Q11). Implementation strategies were captured through an open-ended question (Q12). Coaches were also asked whether they obtain IPP information from social media, and which platforms (e.g., YouTube, Instagram, Facebook, TikTok, X/Twitter) they use (Q10). Respondents could select multiple options but were asked to indicate one primary source.

(iv) Perceptions of IPPs—Coaches who reported using IPPs were presented with five statements regarding the effects of IPPs (Q13). Using a 5-point Likert scale (1 = Strongly disagree, 5 = Strongly agree), they rated the following beliefs: whether IPPs prevent injuries, accelerate return to sport, improve fitness, enhance joint range of motion, or increase muscle tension.

### 2.3. Statistical Analysis

All statistical analyses were performed using Python (v3.11; Python Software Foundation, Wilmington, DE, USA), with the exception of the Firth logistic regression model, which was implemented in R (v4.3.2; R Foundation for Statistical Computing, Vienna, Austria) using the logistf package to handle the small sample size and potential separation bias.

Categorical variables (e.g., gender, sport type, place of residence, and information source) were summarized using counts and percentages. Continuous variables (e.g., age) were reported as means with standard deviations (SD). Group differences in categorical variables were tested using chi-square tests of independence. Where expected cell frequencies fell below five, Fisher’s exact test was applied. Effect sizes for chi-square tests were estimated using Cramér’s V. For comparisons involving continuous or ordinal variables (e.g., Likert-scale responses, age), non-parametric Mann–Whitney U tests and Kruskal–Wallis H tests were used, depending on the number of comparison groups. Associated effect sizes were calculated as the rank-biserial correlation coefficient (r) for U tests and η^2^ for H tests. Effect sizes are interpreted as small (r ≈ 0.1/η^2^ ≈ 0.01), medium (r ≈ 0.3/η^2^ ≈ 0.06), and large (r ≥ 0.5/η^2^ ≥ 0.14).

Two-proportion z-tests were used to compare IPP usage rates between key subgroups, with exact binomial 95% confidence intervals reported for implementation rates. Differences in median perceptions were tested across gender and information source groups.

To identify predictors of IPP usage among those aware of such programs, Firth’s penalized logistic regression was applied. Predictor variables included age, gender, type of sport, place of residence, and source of information. Odds ratios (ORs), 95% confidence intervals (CIs), and *p*-values were reported. Model diagnostics confirmed good fit and the absence of convergence issues. Finally, PAM clustering using Gower distance was performed to identify coach profiles based on mixed-type variables (categorical, ordinal, or binary). The optimal cluster number was determined using silhouette width. Surveys with incomplete responses were excluded from analysis during the eligibility screening phase. Only fully completed questionnaires were retained for statistical analysis. All significance thresholds were set at α = 0.05.

## 3. Results

### 3.1. Participants

Of the 119 completed surveys, 108 responses met the inclusion criteria. Ten participants were excluded due to working exclusively with adult athletes (>18 years old), while one was excluded due to incomplete data. The majority resided in cities with populations up to 500,000. Coaches represented various sports, with soccer, volleyball, handball, and basketball being the most common. Other sports included tennis (*n* = 10; 9.3%), table tennis (*n* = 7; 6.5%), and rugby (*n* = 3; 2.8%), with one coach reporting that they coach karate (*n* = 1; 0.9%). The age distribution of participants was as follows: 27 coaches were under 30 years of age, 43 were aged 30–39, 23 were 40–49, 12 were 50–59, and 3 were aged 60 or above. Full demographic details are presented in Table 1a.

### 3.2. Awareness and Usage of IPPs

Among all participants, 59 coaches (54.6%) reported being aware of IPPs in youth sports, while 49 (45.4%) were not. The most frequently cited sources of IPP knowledge were formal training sessions and fellow coaches. Despite this awareness, only 28 of the 59 informed participants (47.5%) reported actively using IPPs in their coaching practice, whereas 31 (52.5%) did not.

Binomial proportion tests indicated no statistically significant differences in the proportions of respondents aware of IPPs (*p* = 0.334, 95% CI: [0.452, 0.637]) or implementing them (*p* = 0.696, 95% CI: [0.353, 0.600]).

Among IPP users, the most frequently implemented programs included the OSTRC (*n* = 8; 28.6%) and FIFA 11+ (*n* = 7; 25.0%). Six participants (21.4%) used multiple programs (e.g., VolleyVeilig, PEP, Touchfit360, and FootyFirst), and three (10.7%) reported using non-scientific programs not listed in the survey. Two participants (7.1%) did not specify the program used. In terms of integration into coaching routines, 18 participants (72.0%) reported applying IPPs as part of warm-up exercises. Only three participants implemented IPPs one to three times per week outside of warm-up contexts.

### 3.3. Demographic Correlates of IPP Awareness and Usage

Younger coaches were more likely to be aware of IPPs (U = 1092.0, *p* = 0.029, r = −0.210), indicating a small effect size. However, among those aware of IPPs, no significant age differences were found between those who implemented such programs versus those who did not (U = 385.5, *p* = 0.466, r = −0.096).

No significant associations were found between gender, place of residence, or sport type and either awareness or use of IPPs. Full statistical comparisons are detailed in Table 1a,b.

### 3.4. Influence of Information Source on Implementation

**Figure 1:**
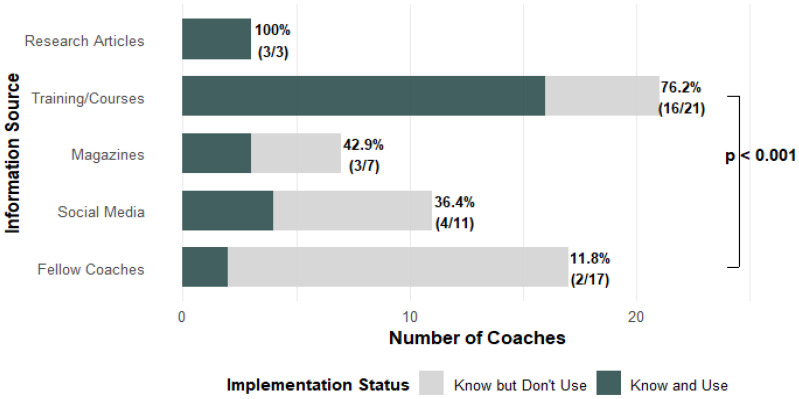
IPP awareness vs. implementation by information source.

**Figure 2:**
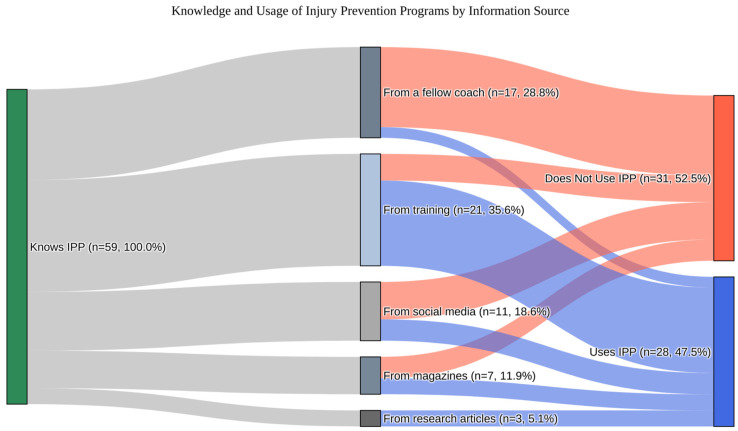
Sankey diagram: awareness of IPPs → source of information → implementation.

A significant association was observed between the source of information and implementation of IPPs (χ^2^ = 21.325, *p* < 0.001). Coaches who learned about IPPs through formal training or courses were substantially more likely to use them compared to those who received information from fellow coaches. Specifically, 76.2% of coaches informed via training or courses (16 out of 21) implemented IPPs, while only 11.8% of those informed by fellow coaches (2 out of 17) reported doing so (Figure 1). This 64.4% difference in implementation rate was statistically significant based on a two-proportion z-test (*Z* = 3.955, *p* < 0.001; 95% CI: 0.406 to 0.882). The same effect was confirmed by an additional chi-square test (*χ*^2^ = 13.164, *p* < 0.001). Figure 2 illustrates that the pathway from IPP awareness to implementation depends on the information source, with formal training leading to markedly higher uptake than peer-based channels.

### 3.5. Predictive Factors for IPPs’ Usage

To address the relatively small sample size and potential separation bias, a Firth’s penalized logistic regression model was applied to identify predictors of IPP implementation among coaches who were aware of such programs (*n* = 59).

The analysis identified information source as the only statistically significant predictor of IPP usage (*p* ≤ 0.001). Coaches who received information through formal training had 20.36 times higher odds of implementing IPPs compared to those informed by fellow coaches (*p* < 0.001), while those citing research articles had 34.47 times higher odds (*p* = 0.012). Although magazines (OR = 4.41) and social media (OR = 3.52) were associated with elevated odds, these effects were not statistically significant (*p* > 0.05).

No significant associations were found for other demographic or contextual variables, including age (OR = 0.99, *p* = 0.805), gender (OR = 0.60, *p* = 0.548), sport coached (*p* = 1.000), or place of residence (*p* = 0.720). Model diagnostics indicated good fit (log-likelihood: −25.927 vs. null model: −35.985), with no evidence of multicollinearity or convergence issues. Full regression results are presented in Table 2, and expanded coefficient-level details are available in Appendix A Table A1.

### 3.6. Perceptions of IPPs’ Impact

**Figure 3:**
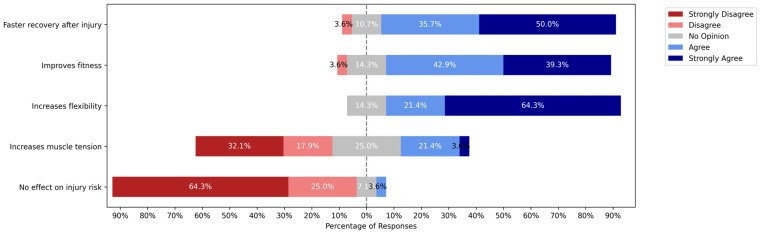
Perceptions of IPPs’ impact among coaches who use IPPs (*n* = 28). Legend: Stacked bar chart showing agreement levels across domains: injury reduction, recovery, fitness, flexibility.

Among the 28 coaches who reported using IPPs, perceptions varied across key domains (Figure 3). A majority (64.3%) strongly agreed that IPPs help to reduce the number of injuries, although a notable proportion expressed skepticism: 25.0% disagreed and 3.6% strongly disagreed. In terms of recovery facilitation, 50.0% strongly agreed that IPPs accelerate return to sport following injury, and 35.7% agreed. Only 3.6% disagreed, while 10.7% remained neutral. Regarding fitness enhancement, 39.3% strongly agreed and 42.9% agreed that IPPs improve general fitness and endurance. Meanwhile, 3.6% disagreed and 14.3% had no opinion. Most coaches (64.3%) strongly agreed that IPPs increase joint flexibility, with an additional 21.4% agreeing. In contrast, 17.9% disagreed and 14.3% expressed no opinion on this item.

### 3.7. Differences in Perceptions by Gender and Information Source

Non-parametric comparisons revealed no statistically significant gender-based differences in coaches’ perceptions of IPPs across any measured domain (*all p* > 0.05, Mann–Whitney *U* test) (Table 3). However, the belief that IPPs accelerate recovery approached significance (*U* = 48.0, *p* = 0.079), with a medium effect size (*r* = −0.308), suggesting a potential trend worth investigating in future research. Similarly, Kruskal–Wallis tests found no significant differences in perceived IPP effectiveness based on the source of information (*all p* > 0.05). Meanwhile, a large effect size emerged for the perception that IPPs improve recovery (*H* = 9.179, *p* = 0.057, η^2^ = 0.225), again indicating a trend that may reach significance with a larger sample.

### 3.8. Cluster Analysis of Coach Profiles

To explore patterns in coach characteristics and beliefs regarding IPPs, clustering analysis was conducted using the subset of participants who both knew and implemented IPPs and who had complete data (*n* = 28) (Table 4). A Gower distance matrix was computed to accommodate mixed data types (categorical, ordinal, binary). Partitioning Around Medoids (PAM) clustering was applied with candidate cluster solutions ranging from *k* = 2 to *k* = 7. The optimal number of clusters was determined to be three, based on the highest silhouette score (0.3502).

Cluster distribution was as follows: Cluster 0, *n* = 4; Cluster 1, *n* = 17; and Cluster 2, *n* = 7. All clustered coaches reported awareness and use of IPPs, allowing the clustering to focus on beliefs, source of knowledge, gender, and sport context (Table 4). Together, these clusters reflect three distinct user profiles: (0) ambivalent implementers with balanced gender representation and mixed opinions; (1) highly enthusiastic adopters, strongly aligned with formal education and favoring IPPs broadly; and (2) cautiously supportive users, predominantly male football coaches with moderate perceptions.

## 4. Discussion

This study aimed to evaluate the awareness, use, and beliefs regarding IPPs among youth sports coaches in Poland and to identify the factors influencing their implementation. This topic is especially important in the context of increasing youth sports dropout rates (particularly among females) [17], where health promotion and injury prevention remain underdeveloped areas in policy and practice [18,19]. A recent systematic review of 19 studies summarized the general deficiencies in coaches’ knowledge about IPPs [12]. Our results align with previous findings from Donaldson et al. (2018), who reported that 42% of soccer coaches were unaware of the FIFA 11+ program, and only 31% implemented it [7].

Younger coaches were more likely to be aware of IPPs (*p* = 0.029), possibly reflecting greater exposure to contemporary training. However, age did not differentiate users from non-users among those already aware of IPPs (*p* = 0.466). A significant association was found between the source of information and IPP implementation. Coaches who learned about IPPs through formal training had a much higher adoption rate (76.2%) than those who learned about them from peers (11.8%). This pattern was confirmed by regression analyses, where information source was the only significant predictor (OR = 3.52–34.47, *p* ≤ 0.001). Other variables—including age, gender, sport coached, and place of residence—were not significant. The lack of association between sport type and IPP usage may be explained by the fact that most popular youth team sports (e.g., soccer, handball, basketball, volleyball) share similar training structures [20]. As such, differences in implementation are less likely to stem from sport-specific culture and more from individual coach exposure and education [21]. Overall, coaches who received a structured education (e.g., courses or scientific studies) were significantly more likely to implement IPPs, reinforcing earlier findings [13]. These usage patterns raise important questions about the underlying beliefs coaches hold regarding the effectiveness of IPPs.

### 4.1. Beliefs and Perceived Effectiveness of IPPs

To better understand why awareness and use remain limited, we explored coaches’ perceptions of the benefits of IPPs. Consistent with previous studies reporting generally positive attitudes toward IPPs among coaches [12,14,22], our findings suggest a broadly supportive stance toward the perceived effectiveness of IPPs. A majority strongly agreed that these programs aid in faster recovery and improved fitness. However, belief in injury reduction—arguably the core aim of IPPs—was not as strong as expected: only 64.3% strongly agreed, while 25% disagreed and 3.6% strongly disagreed that IPPs reduce injury risk. This level of skepticism is notably higher than that reported by Donaldson et al. (2018), where only 6% of coaches rated the FIFA 11+ program as “very or somewhat ineffective” and 15% were “unsure” of its benefits [7]. This discrepancy raises questions about the translational gap between scientific evidence and practical experience. While the efficacy of IPPs has been widely documented in controlled trials, coaches may be unconvinced of their real-world utility, especially when outcomes are not immediately observable, or if injuries still occur despite program use [23]. This creates a paradox: although many coaches express belief in the value of IPPs, only a fraction actually implement them. This gap between belief and behavior is a well-documented challenge in the implementation of evidence-based interventions [24].

A similar pattern was observed in the perception of joint flexibility benefits: although most coaches agreed with this statement, a notable proportion remained neutral or disagreed, indicating mixed confidence in this domain. These findings echo results from a Swedish study, where amateur football coaches who implemented IPPs during the season expressed greater confidence in their injury prevention knowledge than those who had not used such programs [25].

Previous studies found that coaches who are positive about IPPs may have doubts about their implementation in real training environments [12,14]. This mismatch between general approval and real-world execution underscores the need for stronger education, tailored guidance, and clearer evidence of effectiveness in coaches’ day-to-day environments [26]. However, not all coaches who implement IPPs fully trust in their effectiveness. To unpack this further, we analyzed clusters of belief patterns and implementation behavior.

### 4.2. Coach Profiles and Belief Patterns

Cluster analysis revealed three distinct profiles among coaches who used IPPs. Cluster 0 (basketball coaches, gender-balanced) showed ambivalence—despite using IPPs, half expressed no opinion or disagreement about their preventive effects. We define “ambivalent implementers” as coaches who apply IPPs but lack strong beliefs in their benefits, indicating uncertainty or passive compliance rather than conviction. Cluster 1 (mainly male football and volleyball coaches) had the strongest support for IPPs, with most receiving information from formal training and expressing consistent agreement with their benefits. Cluster 2 (male football coaches) showed moderate support but more uncertainty, particularly regarding flexibility and muscle tension. These “cautiously supportive users” are coaches who implement IPPs with some belief in their value but remain skeptical about certain outcomes, reflecting partial buy-in. These profiles underscore how formal education fosters both adoption and belief, whereas informal channels are linked with doubt [27].

### 4.3. Limitations

This study has several limitations that should be acknowledged. First, the use of a web-based survey may have introduced selection bias, as only coaches with access to digital platforms and those who were willing to participate online were included, potentially limiting the generalizability of the findings. The recruitment method, which relied heavily on social media and direct email outreach, may also have resulted in an overrepresentation of coaches who are more engaged with technology and online resources. Notably, 88.9% of respondents were city-based, which may reflect a sampling bias or uneven access to structured training opportunities. Furthermore, the relatively small sample size, particularly for certain sports categories, limits the statistical power to detect significant associations between demographic variables and the use of IPPs. This study did not compute internal consistency for Likert-scale items, as they were not intended to measure a single latent construct. Future studies using psychometric scaling should consider assessing reliability using Cronbach’s alpha. Additionally, the use of Likert-scale questions may have been influenced by response biases such as acquiescence bias (the tendency to agree with statements) and social desirability bias, where participants may provide answers they believe are more socially acceptable rather than their genuine opinions. Moreover, as this study relied entirely on self-reported data, there is a risk of discrepancy between coaches’ stated attitudes or behaviors and their actual practice, particularly regarding the implementation of IPPs. Lastly, this study’s cross-sectional design limits our ability to draw causal inferences about the relationship between IPP use and outcomes.

### 4.4. Practical Implications

In Poland, as in many countries, the use of IPPs is not mandatory [28], hence it is up to the respective associations, individual clubs, and coaches to decide on their implementation in practice [29,30]. Our findings highlight the critical role of continued coach education—especially through formal training and courses—in driving IPP adoption. Doubts regarding injury reduction benefits may be a key barrier and should be addressed through targeted communication and updated scientific evidence. Strengthening coaches’ belief in the effectiveness of IPPs and simplifying their integration into regular training should be priorities for both educational practice and future research. To close the persistent gap between knowledge and implementation, it is essential to expand structured training access, reinforce evidence-based confidence in IPPs, and tailor implementation strategies to the specific needs of Polish coaches.

## 5. Conclusions

The implementation of IPPs among youth sports coaches in Poland remains limited, with only 47.5% of aware coaches applying them. Formal education was the strongest predictor of use, while demographic and sport-related factors were not significant. These results support the need for structured strategies, such as mandatory coach education, accessible workshops, and online training tools. Though based in Poland, the findings may apply to other countries with similar barriers. Limitations include non-probability sampling and reliance on self-report. Future research should explore implementation behaviors and evaluate long-term impact using longitudinal or interventional studies.

## Figures and Tables

**Table 1:** (**a**) Demographic characteristics of coaches (*n* = 108) by IPP awareness. (**b**) Demographic characteristics among coaches who were aware of IPPs (*n* = 59).

**(a)**					
**Variable**	**Category**	**Total *n* (%)**	**Aware of IPPs *n* (%)**	**Not Aware of IPPs *n* (%)**	**χ^2^/*p*/Cramér’s V**
Gender	Male	76 (70.4%)	40 (37.0%)	36 (33.3%)	χ^2^ = 0.186, *p* = 0.666, V = 0.0
	Female	32 (29.6%)	19 (17.6%)	13 (12.0%)	
Place of Residence	Village	12 (11.1%)	6 (5.6%)	6 (5.6%)	χ^2^ = 0.704, *p* = 0.703, V = 0.0
	City ≤ 500k	77 (71.3%)	44 (40.7%)	33 (30.6%)	
	City > 500k	19 (17.6%)	9 (8.3%)	10 (9.3%)	
Sport	Soccer	45 (41.7%)	24 (22.2%)	21 (18.5%)	χ^2^ = 3.966, *p* = 0.265, V = 0.105
	Volleyball	16 (14.8%)	9 (8.3%)	7 (6.5%)	
	Handball	13 (12.0%)	5 (4.6%)	8 (7.4%)	
	Basketball	13 (12.0%)	10 (9.3%)	3 (2.8%)	
(**b**)					
**Variable**	**Category**	**Uses IPPs *n* (%)**		**Does Not Use IPPs *n* (%)**	**χ^2^/*p*/Cramér’s V**
Gender	Male	20 (33.9%)		20 (33.9%)	χ^2^ = 0.083, *p* = 0.773, V = 0.0
	Female	8 (13.6%)		11 (18.6%)	
Place of Residence	Village	3 (5.1%)		3 (5.1%)	χ^2^ = 0.05, *p* = 0.976, V = 0.0
	City ≤ 500k	21 (35.6%)		23 (39.0%)	
	City > 500k	4 (6.8%)		5 (8.5%)	
Sport	Soccer	12 (20.3%)		12 (20.3%)	χ^2^ = 0.711, *p* = 0.871, V = 0.0
	Volleyball	4 (6.8%)		5 (8.5%)	
	Handball	2 (3.4%)		3 (5.1%)	
	Basketball	6 (10.2%)		4 (6.8%)	

Legend: χ^2^ = chi-square value; *p* = *p*-value; Cramér’s V = effect size. Sport includes the four most popular sports.

**Table 2:** Predictors of IPP use (Firth logistic regression model, *n* = 59).

Predictor	Effect	Odds Ratio (95% CI)	*p*-Value
Info Source	Categorical (5 levels)	3.52–34.47	0.001 *
Age	Per 1 year increase	0.99 (0.92–1.06)	0.805
Sport	Categorical (8 levels)	0.06–1.31	0.999
Gender	Male vs. Female	0.60 (0.09–3.02)	0.548
Place of Residence	Categorical (3 levels)	2.29–2.94	0.720

Legend. Odds ratios reflect comparisons with a reference category. The “Effect” column specifies how each predictor was modeled in the regression. Confidence intervals are reported for binary and continuous predictors. Categorical predictors (e.g., Info Source, Sport, Place of Residence) were entered as factor variables, and results were interpreted relative to a reference level (e.g., Fellow Coaches for Info Source). Continuous predictors (e.g., Age) reflect change per unit increase. Binary predictors (e.g., Gender) were coded as binary options (male vs. female). *—*p* < 0.05.

**Table 3:** Group differences in coaches’ perceptions of IPPs by gender and information source.

Belief Item	U (Gender)	*p* (Gender)	r (Gender)	H (Info Source)	*p* (Info Source)	η^2^ (Info Source)
No impact on injuries	90.5	0.5487	0.101	4.539	0.3379	0.023
Faster recovery after injury	48.0	0.0786	−0.308	9.179	0.0568	0.225
Improves fitness	68.5	0.5461	−0.111	6.025	0.1973	0.088
Increases joint flexibility	74.0	0.7421	−0.058	7.451	0.1139	0.150
Increases muscle tension	90.5	0.5993	0.101	4.185	0.3816	0.008

Legend. Mann–Whitney *U* tests compared gender differences (male vs. female); Kruskal–Wallis *H* tests assessed differences by IPP information source (e.g., training, social media, fellow coaches). *r* = rank-biserial effect size; η^2^ = Kruskal–Wallis effect size.

**Table 4:** Cluster characteristics based on PAM clustering with Gower distance (*n* = 28).

Item	Cluster 0 (*n* = 4)	Cluster 1 (*n* = 17)	Cluster 2 (*n* = 7)
Gender (Mode)	50% Male, 50% Female	Male (64.7%) Female (35.3%)	100% Male
Sport (Mode)	100% Basketball	Football (35.3%) Volleyball (23.5%)	Football (85.7%) Handball (14.3%)
Info Source (Mode)	50% Training, 50% Fellow Coach	Training (64.7%) Social media (17.6%)	42.9% Training, 28.6% Magazines
**Belief Items**			
No Impact on Injuries	50% No Opinion, 50% Disagree	100% Strongly Disagree	71.4% Disagree, 14.3% Agree
Faster Recovery	100% Agree	70.6% Strongly Agree, 17.6% Agree	42.9% Agree, 28.6% No Opinion
Improves Fitness	50% Agree, 25% No Opinion	64.7% Strongly Agree, 23.5% Agree	85.7% Agree
Increases Flexibility	100% No Opinion	100% Strongly Agree	85.7% Agree, 28.6% No Opinion
Increases Muscle Tension	75% Agree	52.9% Strongly Disagree, 23.5% No Opinion	42.9% No Opinion, 28.6% Agree

## Data Availability

The original contributions presented in this study are included in the article. Further inquiries can be directed to the corresponding author(s).

## Appendix A

**Table A1:** Individual effects of categorical predictor levels from Firth logistic regression.

Predictor	Logistic Regression Coefficient	Odds Ratio	*p*-Value
From magazines	1.484	4.41	0.219
From research articles	3.540	34.47	0.012 *
From social media	1.259	3.52	0.234
From training	3.014	20.37	<0.001 *
sport_trained Football	−0.350	0.70	0.732
sport_trained Handball	−0.345	0.71	0.781
sport_trained Karate	−2.762	0.06	0.111
sport_trained Rugby	0.269	1.31	0.893
sport_trained Table Tennis	−1.202	0.30	0.474
sport_trained Tennis	−0.096	0.91	0.937
sport_trained Volleyball	−0.230	0.79	0.839
City up to 500K	1.079	2.94	0.266
Village	0.827	2.29	0.598

Legend. *—*p* < 0.05.

## Appendix B

**Table A2:** Web-based survey for youth sports coaches.

No.	Question	Response Options
1	Do you consent to participate in this scientific study?	☐ Yes ☐ No
2	Gender	☐ Female ☐ Male ☐ Non-binary ☐ Prefer not to say
3	Age	……………………………………………
4	Size of place of residence	☐ City over 500,000 inhabitants ☐ City 150,000–500,000 ☐ City 50,000–150,000 ☐ Town under 50,000 ☐ Village
5	Do you coach a youth sports team aged 9–17 years?	☐ Yes ☐ No
6	What sport do you coach?	☐ Football ☐ Volleyball ☐ Handball ☐ Basketball ☐ Tennis ☐ Table Tennis ☐ Rugby ☐ Other: …………………
7	Are you familiar with injury prevention programs (IPPs) for the musculoskeletal system?	☐ Yes ☐ No
8	How did you learn about injury prevention programs?	☐ From training/workshop ☐ From social media ☐ From scientific articles ☐ From magazines (print/online) ☐ From a coaching peer ☐ Other: …………
9	Do you use injury prevention programs in your team?	☐ Yes ☐ No
10	Do you get information about IPPs from social media? If so, which ones?	☐ YouTube ☐ Instagram ☐ Facebook ☐ TikTok ☐ X (Twitter) ☐ No
11	Which IPPs do you use?	☐ FIFA 11+ ☐ OSTRC ☐ ISPRINT ☐ Touchfit360 ☐ PEP ☐ FootyFirst ☐ Aspetar Hamstring Protocol ☐ Aspetar Adductor Protocol ☐ VolleyVeilig ☐ None ☐ Other: …………
12	How do you implement injury prevention programs in your practice?	……………………………………………………………………………………………………………
13	Please respond to the following statements by selecting your level of agreement:	Scale: 1—Strongly disagree, 2—Rather disagree, 3—Neutral, 4—Rather agree, 5—Strongly agree
Statement items for Question 13: 1. Injury prevention programs have no effect on injury occurrence. 2. IPPs accelerate return to sport after injury. 3. IPPs increase athletes’ fitness and endurance. 4. IPPs improve joint range of motion. 5. IPPs cause increased muscle tension.		
